# Single-Cell Analysis of Contractile Forces in iPSC-Derived Cardiomyocytes: Paving the Way for Precision Medicine in Cardiovascular Disease

**DOI:** 10.3390/ijms241713416

**Published:** 2023-08-30

**Authors:** Irene C. Turnbull, Apratim Bajpai, Katherine B. Jankowski, Angelo Gaitas

**Affiliations:** 1 Cardiovascular Research Institute, Icahn School of Medicine at Mount Sinai, New York, NY 10029, USA; 2 The Estelle and Daniel Maggin Department of Neurology, Icahn School of Medicine at Mount Sinai, New York, NY 10029, USA; 3 BioMedical Engineering & Imaging Institute, Leon and Norma Hess Center for Science and Medicine, New York, NY 10029, USA

**Keywords:** single-cell mechanics, single-cell lateral force, single-cell normal force, force–frequency relationship, normal and lateral force correlation, contractile force, force dynamics, induced pluripotent stem cell-derived cardiomyocytes (iPSC-CMs), Atomic Force Microscopy (AFM)

## Abstract

Induced pluripotent stem cell-derived cardiomyocytes (iPSC-CMs) hold enormous potential in cardiac disease modeling, drug screening, and regenerative medicine. Furthermore, patient-specific iPSC-CMS can be tested for personalized medicine. To provide a deeper understanding of the contractile force dynamics of iPSC-CMs, we employed Atomic Force Microscopy (AFM) as an advanced detection tool to distinguish the characteristics of force dynamics at a single cell level. We measured normal (vertical) and lateral (axial) force at different pacing frequencies. We found a significant correlation between normal and lateral force. We also observed a significant force–frequency relationship for both types of forces. This work represents the first demonstration of the correlation of normal and lateral force from individual iPSC-CMs. The identification of this correlation is relevant because it validates the comparison across systems and models that can only account for either normal or lateral force. These findings enhance our understanding of iPSC-CM properties, thereby paving the way for the development of therapeutic strategies in cardiovascular medicine.

## 1. Introduction

Induced pluripotent stem cells (iPSCs) are pluripotent stem cells generated from adult cells, such as adult human skin fibroblasts or peripheral blood mononuclear cells [1], through reprogramming [2]. These iPSCs can then be differentiated into various cell types, including cardiomyocytes (CMs) [2]. iPSC-CMs have significant potential in a variety of fields, including cell therapy, drug assessment, and the study of cardiac diseases’ genetic and pathophysiological mechanisms [3,4,5,6]. In particular, iPSC-CMs are emerging as a promising tool for evaluating drugs, with the potential to predict patient-specific cardiotoxicity and aid in personalized medicine [7,8,9]. Given that approximately one third of all drugs have cardiovascular side effects, the use of iPSC-CMs as a human cellular model for predicting drug arrhythmogenicity is particularly crucial [10,11,12]. iPSC-CMs in combination with extracellular matrix components are employed for the fabrication of engineered cardiac tissues in a multitude of shapes and for various applications [13,14,15,16,17], showing promising results in regenerative medicine, as, in the future, they can be used to repair or replace damaged heart tissue [18,19]. Therefore, as the technology continues to advance, iPSC-CMs may play a significant role in the development of engineered tissue and personalized medicine.

The advancement of the clinical application of iPSC-CMs for drug discovery and reparative therapeutic strategies relies heavily on the ability to properly characterize iPSC-CMs at a single-cell level. In this context, a comprehensive characterization of these cells, particularly their contractile force dynamics, is crucial [20,21,22,23,24,25,26,27,28]. Additionally, the application of iPSC-CMs for drug toxicity screening necessitates innovative tools. Atomic Force Microscopy (AFM) is a high precision tool used to investigate the forces exerted by contracting iPSC-CMs [20,21,22,23,24,25,26,27,28]. Two forces, namely vertical (normal) and horizontal (lateral–axial), are of prime interest. While Atomic Force Microscopy (AFM) has traditionally been used to measure vertical forces, lateral forces offer insight into axial behavior. Despite their significance, the correlation between these forces remains poorly understood [29]. This study investigates the relationship between vertical (normal) and axial (lateral) forces exerted by iPSC-CMs at different pacing frequencies, providing a novel perspective on the mechanistic behavior of these cells and opening new avenues for future research in cardiac therapeutics.

## 2. Results

### 2.1. AFM Measurements Distinguish Normal and Lateral Force from Single-Cells

**Figure 1:**
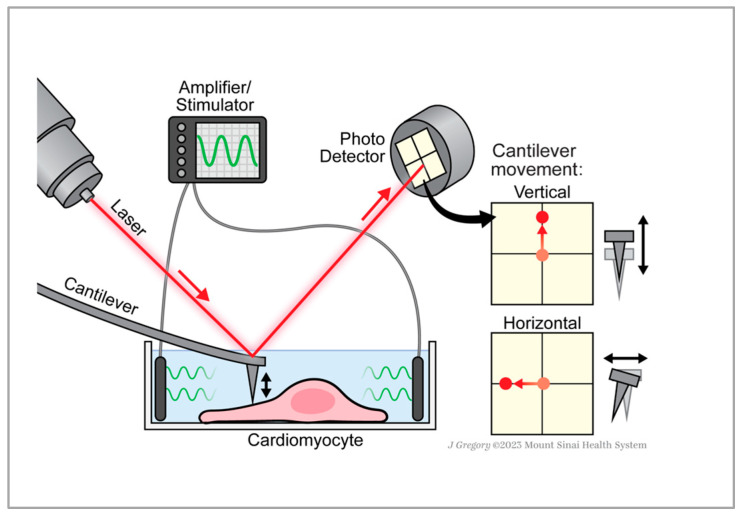
Experimental setup for normal and lateral force monitoring. Schematic of AFM experimental setup (not to scale), illustrating how normal (vertical) and lateral (horizontal) forces are recorded. The laser’s deflection off the cantilever’s backside is captured by the photodetector. The vertical motion of the cantilever, representing the normal force, corresponds to the vertical movement of the laser on the detector. Conversely, the cantilever’s lateral twist, or sideways motion, representing the lateral force (bottom panel), is mirrored by the laser moving left and right. Arrows indicate the direction of laser movement. For electrical stimulation, a pair of carbon electrodes are connected to an amplifier/stimulator. Printed with permission from ©Mount Sinai Health System.

**Figure 2:**
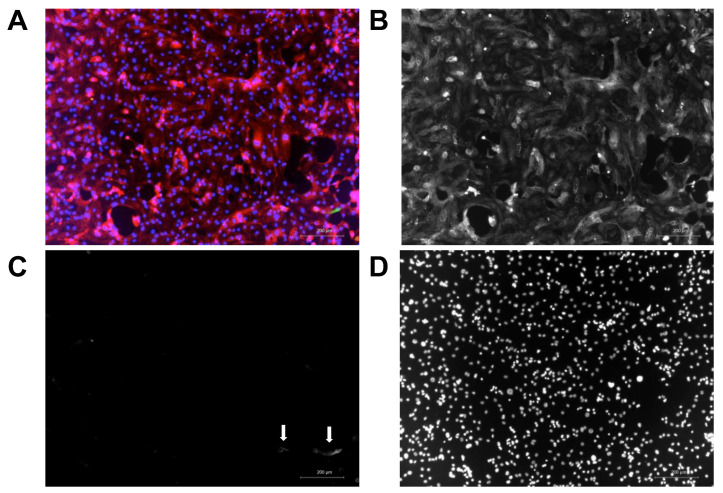
Immunofluorescence of iPSC-CMs. (**A**) Representative immunofluorescence image of iPSC-CM (aged matched to the iPSC-CM used for AFM force measurements). The cells were stained for the ventricular marker MLC2V (red) and the atrial marker MLC2a (green), the nuclei were counterstained with DAPI (blue). (**B**–**D**) Gray scale images of each individual channel showing that the cells consistently express the ventricular marker MLC2V (**B**, red channel), with only a few cells displaying the atrial marker MLC2A (**C**, green channel, arrows point to cells); blue channel shown in (**D**). Scale bar 200 µm.

AFM methods allow for the evaluation of the vertical force exerted by an individual cell [21], while systems that mimic the 3-dimensional physiological operations of the heart, such as engineered cardiac tissues on flexible posts, are capable of measuring the axial force [13]. This poses an important question: How do these two different force measurements correlate? In past investigations, we aimed to answer this question [30] by using engineered cardiac tissue and specially designed micro-fabricated force sensors. In this study, we established a correlation between the normal and lateral forces at a single-cell level by directly measuring the normal (vertical) force and using the lateral force as a stand-in for the axial force (Figure 1). The cells included in this study were all from the same cell line, but each derived from iPSC-CM differentiations carried out on different dates (three independent differentiation batches). The cells, although heterogeneous in size and morphology on the day of measurements, were consistent in the expression of the ventricular marker myosin light chain 2 (MLC2V) (Figure 2). 

**Figure 3:**
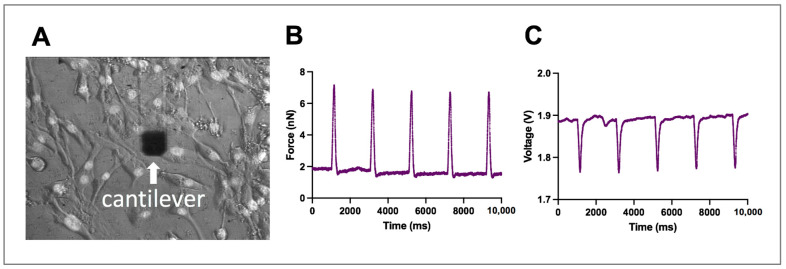
Simultaneous measurement of normal and lateral force. (**A**) Screenshot of field of view of AFM cantilever and iPSC-CMs for normal and lateral force recording. The nuclei were stained with Hoechst; image shown in grayscale. The cantilever is made of SiO_2_ which is transparent to light, except for the dark section that includes a thin metal film for laser reflection. (**B**,**C**) Representative graphs of normal (**B**) and lateral (**C**) force, obtained from iPSC-CM during pacing at 0.5 Hz.

We measured normal and lateral forces from three iPSC-CMs at three different frequencies: 0.5 Hz, 0.7 Hz, and 1 Hz. The normal force of contraction and the lateral force were recorded simultaneously. The AFM cantilever was positioned to target the cytoplasmic region of the beating cell, specifically aligned to face the cell’s most elongated section in order to maximize the lateral movement (Video S1, Figure 3A). For the analysis, first, we confirmed that the cells were beating at each of the prescribed frequencies accordingly. Then, we calculated the average force values from five sequential peaks, determining each contraction’s force value by subtracting the baseline from the peak. The units for the normal force were expressed in nN, and for the lateral force in Voltage (Figure 3B,C). 

### 2.2. Normal and Lateral Force Correlation and Frequency Dependence

**Figure 4:**
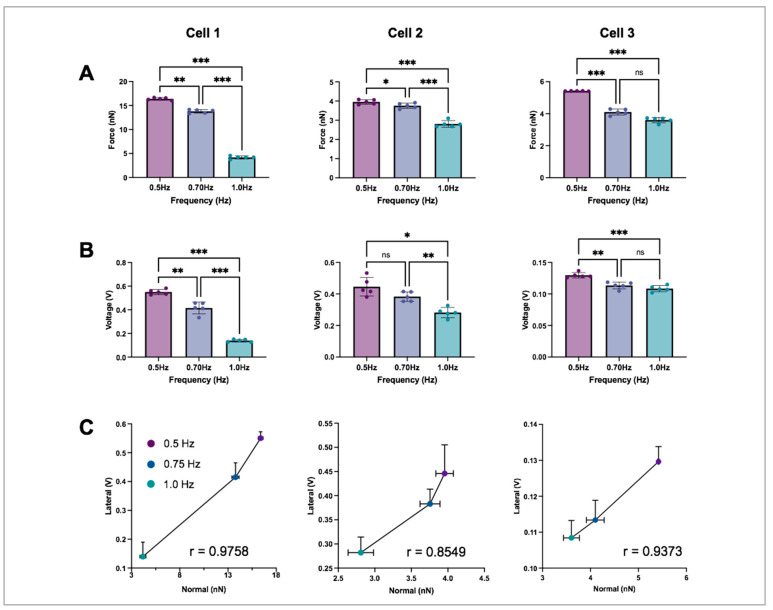
iPSC-CM responses under different pacing frequencies. Variation of normal force (**A**), and lateral force (**B**) measured by the microcantilever with increasing pacing frequency for each cell. Pairwise comparison using repeated measures ANOVA; ns = not significant, * *p* < 0.05, ** *p* < 0.01, *** *p* < 0.001; n = 5 per frequency. (**C**) Pearson correlation coefficient between normal and lateral forces. Values compared at each of the pacing frequencies (0.5 Hz, 0.7 Hz, and 1.0 Hz).

The normal forces we measured ranged from 3 to 16 nN, aligning with the range reported in previous studies [21,31,32,33,34,35]. These calculations were performed using a Python script. We observed that the magnitude of the normal force measured by the microcantilever decreased with increasing frequency; this force–frequency dependence is expected and explained by the innate immaturity of these cells [36,37] (Figure 4A). A similar trend was observed for the lateral force (Figure 4B). In all cases, the values of force were significantly lower at 1.0 Hz compared to the baseline at 0.5 Hz. For cell 1, there was a 75% decrease in force between 0.5 Hz and 1.0 Hz for normal and lateral force. For cell 2, there was a 29% and 37% decrease in normal and lateral force, respectively, between 0.5 Hz and 1.0 Hz. For cell 3, there was a 33% and 16% decrease in normal and lateral force, respectively, between 0.5 Hz and 1.0 Hz. We found a significant correlation between the lateral and normal forces across the cells, with Pearson correlation r values of 0.9758, 0.8549 and 0.9373 (*p* < 0.0001 for each of the three cells) (Figure 4C).

## 3. Discussion

In this study, we evaluated the contractile force of individual iPSC-CMs at different pacing frequencies. One emphasis of this work is on measuring normal and lateral forces, which were concurrently recorded using the same AFM cantilever, in order to investigate the correlation between these two forces. Normal (vertical) forces and lateral (axial) forces can be measured with an AFM (Figure 1). Normal forces in AFM are those that occur perpendicular to the sample surface [38]. These forces are responsible for the vertical deflection of the cantilever. In our case, we do not raster the tip, we measure these forces at a point (without moving the tip) to determine the movement of each cell. Lateral forces, on the other hand, are those that occur parallel to the sample surface. These forces cause a torsion or twisting of the cantilever. Lateral Force Microscopy (LFM) [39] is used to measure these forces, which in our case, can provide information about the in-plane movement of the surface of the cells. In these experiments, we have not calibrated the lateral forces; therefore, we measure the photodetector voltage reading as an approximation of the lateral forces. 

In a prior study [30], we applied an established method for the evaluation of axial force of cardiac tissues engineered from human-induced pluripotent stem cell-derived cardiomyocytes (hiPSC-CMs). The method entailed a device that includes a tissue strip fixed between two flexible polydimethylsiloxane posts. Axial force readings were obtained through the optical tracking of post deflections, from which we derived a value for the active contractile force. Concurrently, we measured the vertical force using a micromachined cantilever force sensor, specifically designed to touch and record tissue contractions. We identified a strong correlation between the two forces; however, it was unclear if this correlation would persist at a single-cell level in hiPSC-CMs. Recognizing the importance of the study of single-cell mechanics, other AFM-based approaches have been employed to interrogate cardiomyocyte contractile force at a single cell level in combination with other techniques. The axial and vertical forces were measured simultaneously by combining AFM and carbon fiber techniques on isolated mouse ventricular cardiomyocytes [40]. The cardiomyocytes during contractions displayed positive vertical force and negative axial force, attributed to cell volume redistribution [40]. Axial forces and vertical forces were evaluated in isolated adult rat ventricular cardiomyocytes, and compared to changes in cellular calcium concentration using fluorescence confocal scanning microscopy; it was reported that the axial (torsional) signal displayed a correlation with changes in calcium concentration [41]. 

Here, we demonstrated a correlation of normal and lateral force. We also observed that variation in the amount of force between individual cells. Cardiomyocytes exhibit variations in the normal and lateral forces they generate due to a myriad of factors. One of the primary determinants is the cellular structure and composition of the cardiomyocytes. The arrangement, length, and density of sarcomeres, which are the basic contractile units within these cells, play a pivotal role [42]. Furthermore, the relative composition of myofilaments, including actin and myosin, can significantly influence the contractile force [43]. The size and morphology of cardiomyocytes also matter; for instance, a longer cardiomyocyte with more sarcomeres in series may generate a different force compared to a wider cell with sarcomeres arranged in parallel [44,45]. Beyond these intrinsic properties, the type of cardiomyocyte—whether atrial, ventricular, or nodal—also introduces variability in force dynamics [46]. The metabolic state of the cell, especially the availability of energy molecules such as ATP, directly impacts its contractility [47]. Environmental factors, such as the cell’s interaction with the surrounding extracellular matrix (ECM) and neighboring cells, can modulate contraction forces [48]. A stiffer ECM might amplify the contractile response, while close cell-to-cell interactions can introduce variations compared to isolated cells [49]. Another crucial factor is ion homeostasis. Calcium ions, in particular, govern the contraction of cardiomyocytes, and any variation in calcium handling can lead to differences in force generation between cells [50]. Intracellular signaling pathways, especially those influenced by external stimuli such as beta-adrenergic stimulation, can further modulate the force dynamics [51]. Pathological conditions introduce another layer of complexity. For example, cardiomyocytes from hearts with conditions such as hypertrophy or dilation may behave differently due to structural, metabolic, and signaling than cardiomyocytes from healthy hearts [52]. Lastly, the age and maturation status of the cardiomyocyte can introduce variability, with younger, less mature cells exhibiting different contractile properties than their mature counterparts [53]. In summation, the force variations observed between individual cardiomyocytes arise from a complex interplay of cellular, molecular, and environmental factors, underscoring the multifaceted nature of cardiac cell physiology.

In future investigations that utilize hiPSC-CMs as human models, discerning mechanical characteristics at a single-cell level could yield valuable insights into cellular responses to molecular changes and pharmacological interventions. An advantage of our approach is that it can be easily implemented with any existing AFM. Our configuration requires only minimal modifications to incorporate electrical stimulation, achieved by incorporating carbon electrodes connected to a stimulator. 

In conclusion, here we discerned a notable correlation between the normal (vertical) and lateral (axial) forces exhibited by iPSC-CMs across various pacing frequencies measured with an AFM. This finding, in conjunction with our earlier results [30], implies that normal (vertical) force might be effectively used as an indicator for lateral (axial) force at the single cell level to assess cardiomyocyte contractile performance.

## 4. Materials and Methods

### 4.1. Cell Source and Differentiation

The cells used in this study are hiPSCs from a healthy cell line (SKiPS-31.3) [54]. The iPSCs were expanded in StemFlex media on 6-well tissue culture treated plates coated with hESC-qualified Matrigel, in a 5% CO_2_ incubator at 37 °C. iPSCs were differentiated into CMs by following a monolayer-based differentiation protocol with minor modifications [29,55,56]. Briefly, when iPSCs reached about 80% confluency, StemFlex media was replaced with basal medium (RPMI 1640 media plus B27 supplement minus Insulin and 1% penicillin/streptomycin), and 10 μM CHIR99021 for 24 h. On day 1, the medium was replaced with basal medium without CHIR99021 for two days. On day 3, the medium was replaced with basal medium plus 5 μM IWR-1 for another 2 days. On day 5, the medium was replaced with basal medium. On day 7, the cells were supplemented with RPMI 1640 medium plus B27 supplement and 1% penicillin/streptomycin. Afterward, the medium was exchanged every 2 days. Functional iPSC-CMs began to display spontaneous contractions in culture between days 7 and 10 post-CHIR treatment. 

### 4.2. Cell Preparation for AFM Experiments

For the AFM experiments, the iPSC-CMs were maintained in culture for 2 months. Thereafter, the iPSC-CMs were replated onto glass bottom cell culture dishes as follows. First, the glass bottom dishes were Matrigel coated using Matrigel hESC-Qualified Matrix (Corning Catalog #354277) diluted in DMEM/F12 following manufacturer instructions. Immediately after diluting the Matrigel, it was dispensed onto the cell culture dishes ensuring coverage of the bottom surface; the dishes were left undisturbed, inside the biosafety cabinet for one hour. The iPSC-CMs were washed once with PBS without calcium/magnesium, treated with Trypsin-EDTA (0.25%) (1 mL/well) and kept in the incubator (5% CO_2_, 37 °C,) for 7–10 min, monitoring the cells every minute after the first five minutes for detachment; importantly, the plate was transported carefully, without tapping the plate to prevent the cells from detaching and clumping; after the incubation period, the trypsin was removed, whereby only a portion of the cells were removed, with the majority of the iPSC-CMs remaining on the bottom of the dish. Then, the cells were collected from each well by rinsing each well with 1 mL of RPMI 1640 media plus B27 supplement, 1% penicillin/streptomycin, and 10% knockout serum. After gentle pipetting with a P1000 pipette tip, the cells were dispensed into a 50 mL conical tube through a cell strainer (100 µm). From there, the cells were plated onto glass bottom dishes after removing the Matrigel. The following day, the medium was replaced with RPMI 1640 medium plus B27 supplement, 1% penicillin/streptomycin, and changed every other day. The cells displayed spontaneous beating starting on day 3 after replating. The AFM experiments were performed 5–7 days after replating. 

### 4.3. AFM Measurements

We used a FlexAFM (Nanosurf AG, Liestal, Switzerland) with inbuilt temperature controller and an inverted Axio Observer (Carl Zeiss, Jena, Germany) microscope for these measurements with Nanosensors qp-SCONT-10 microcantilever probes with a spring constant of ~0.01 N/m, and tip height of 8 µm. Once the microcantilever made contact with the cell, an AFG-2005 Arbitrary function generator (GW INSTEK) was used to electrically stimulate the cells (Figure 1) with carbon electrodes that were connected to the function generator. The cells were paced at different consecutively increasing frequencies with a 5 V electrical stimulation of 150 µs duration. After a 30-s period for the cells to acclimate to each pacing frequency, this was followed by a 30-s measurement period to collect data on force measurements. The microcantilever was used to measure both normal and lateral forces (Figure 1). The data were extracted with the Nanosurf 1.5.0 python package (Supplementary Materials, Figures S1 and S2). Subsequent data analysis was carried out using MATLAB R2022B (1994–2023 The MathWorks, Inc., Natick, MA, USA).

### 4.4. Immunofluorescence of iPSC-CMs

iPSC-CMs that were age-matched to the iPSC-CMs used for AFM experiments were set aside for immunofluorescence evaluation. The cells were washed with phosphate-buffered saline (PBS) with Ca^2+^/Mg^2+^, then fixed with 4% paraformaldehyde (PFA) for 10 min at room temperature, followed by washing with PBS. For permeabilization, the cells were treated with Triton 0.5% for 10 min, followed by washing with PBS. For blocking, the cells were treated with 10% goat serum for 1 h, followed by incubation with primary antibodies overnight at 4 °C in a humidified chamber. The next morning, the cells were washed with PBS 3 times for 5 min, followed by addition of secondary antibodies and incubation for 2 h at room temperature, in a humidified chamber, protected from light. After washing with PBS 3 times for 5 min, the cells were counterstained with DAPI. Imaging was performed using a Keyence fluorescence microscope BZ-X800E. The primary antibodies used were myosin light chain 2 (MLC-2V) Polyclonal antibody (Proteintech Cat#10906-1-AP, 1:300), and myosin light chain 2 (MLC-2A) mouse monoclonal antibody (Synaptic Systems Cat# 311011, 1:300). The secondary antibodies were Goat anti-Mouse IgG (H+L) Highly Cross-Adsorbed Secondary Antibody, Alexa Fluor Plus 488 (Invitrogen, Cat# A32723, 1:400, Waltham, MA, USA), and Goat anti-Rabbit IgG (H+L) Highly Cross-Adsorbed Secondary Antibody, Alexa Fluor Plus 555 (Invitrogen, Cat# A32732, 1:400). The antibodies were diluted in Dako Antibody Diluent with Background-Reducing Components (Agilent Technologies, Santa Clara, CA, USA).

## Data Availability

The data presented in this study are available on request from the corresponding author.

## Supplementary Materials

The following supporting information can be downloaded at: https://www.mdpi.com/article/10.3390/ijms241713416/s1.
