# Supplementary material for: Single-Cell Analysis of Contractile Forces in iPSC-Derived Cardiomyocytes: Paving the Way for Precision Medicine in Cardiovascular Disease
Source: Int J Mol Sci. 2023 Aug 30;24(17):13416. doi: 10.3390/ijms241713416 (PMC10487756; doi:10.3390/ijms241713416)
Supplement: Supplementary file 1 [file ijms-24-13416-s001.zip › Supplementary Material_IJMS_proofreading.pdf]

Supplementary Materials.

Instructions and Script to extract data from AFM measurements

Step 1: Install PyCharm

PyCharm Community Edition, download and install it from the official JetBrains website.

Step 2: Open PyCharm

Launch PyCharm Community Edition after the installation is complete.

Step 3: Create or Open a Project

Create New Project: Click "New Project" to create a new Python project. Specify the project name and location.

Open Existing Project: If you already have a project, click "Open" and navigate to that project's folder.

Step 4: Add main.py to Project Directory

Drag and drop the main.py file into the PyCharm project directory.

Alternatively, you can manually copy and paste main.py into the project directory using your system's file explorer.

Step 5: Install Required Packages

Open the terminal inside PyCharm (usually at the bottom of the PyCharm window) and install the necessary packages using pip install.

Step 6: Check Run Configuration

Go to the top-right corner of the PyCharm window, find the run configurations dropdown, and click "Edit Configurations" (it looks like a gear icon).

Ensure that the "Script path" points to your main.py file.

Click "Apply" and then "OK".

Step 7: Run the Script

Right-click on the main.py file in the project explorer on the left side of PyCharm and choose "Run 'main'".

Alternatively, you can click the "Run" button at the top-right corner of the PyCharm window.

Script

```
import tkinter.filedialog as filedialog
import tkinter as tk
from NSFopen.read import read as afmreader
from pandas import DataFrame, ExcelWriter
from matplotlib.pyplot import plot, show
from matplotlib.backends.backend_tkagg import FigureCanvasTkAgg
from matplotlib.figure import Figure
from numpy import mean, append
from scipy.signal import find_peaks

global DATA

def data_automation():
    global DATA
    filename = input_entry.get()
    alldata = afmreader(filename, verbose=False).data
    # Reading data
    adhesion_data = alldata['Spec']['Pause Forward']['Deflection']
    friction_data = alldata['Spec']['Pause Forward']['Friction force']
    adhesion_force = adhesion_data[0][int(float(set_range_min.get())) *
                                1000: int(float(set_range_max.get())) * 1000] * 1e9
    friction_force = friction_data[0][int(float(set_range_min.get())) *
                                1000: int(float(set_range_max.get())) * 1000]
    # Saving data
    d = {'Time (sec)': [i / 1000 for i in range(int(float(set_range_min.get())) * 1000,
                                      int(float(set_range_max.get())) * 1000)]
        ,'Adhesion Data': adhesion_force,
         'Friction Data': friction_force}
    df_1 = DataFrame(data=d)
    dict_2 = {"Peak height (nN)": DATA["Adhesion"]["peak_heights"],
             'Peak widths (sec)': DATA["Adhesion"]["peak_widths"] / 1000,
             'Time b/n Pulses (sec)': DATA["Adhesion"]["time_bn_peaks"] / 1000,
             'Peak time (sec)': DATA["Adhesion"]["peaks"] / 1000, }
    df_2 = DataFrame(data=dict_2)

    dict_3 = {"Peak height (mV)": DATA["friction"]["peak_heights"],
             'Peak widths (sec)': DATA["friction"]["peak_widths"] / 1000,
             'Time b/n Pulses (sec)': DATA["friction"]["time_bn_peaks"] / 1000,
             'Peak time (sec)': DATA["friction"]["peaks"] / 1000,}

    df_3 = DataFrame(data=dict_3)
    file_path = filename[0:-4] + "DATA.xlsx"
    file_path.replace("\\", "/")
```

```
    print(file_path)
    writer2 = ExcelWriter(file_path)
    df_1.to_excel(writer2, sheet_name='Raw Data', index=False)
    df_2.to_excel(writer2, sheet_name='Adhesion Force Data', index=False)
    df_3.to_excel(writer2, sheet_name='Friction Force Data', index=False)
    writer2.save()

def input_field():
    global DATA
    input_path = tk.filedialog.askopenfilename()
    input_entry.delete(1, tk.END)  # Remove current text in entry
    input_entry.insert(0, input_path)  # Insert the 'path'
    filename = input_entry.get()
    alldata = afmreader(filename, verbose=False).data
    # Reading data
    adhesion_data = alldata['Spec']['Pause Forward']['Deflection']
    adhesion_force = adhesion_data[0]

    set_range_min.insert(0, '0')
    set_range_max.insert(0, str(len(adhesion_force) / 1000))
    DATA = {}

def start_plot():
    filename = input_entry.get()
    alldata = afmreader(filename, verbose=False).data
    # Reading data
    adhesion_data = alldata['Spec']['Pause Forward']['Deflection']
    friction_data = alldata['Spec']['Pause Forward']['Friction force']
    adhesion_force = adhesion_data[0][int(float(set_range_min.get())) *
                                 1000: int(float(set_range_max.get())) * 1000] * 1e9
    friction_force = friction_data[0][int(float(set_range_min.get())) *
                                 1000: int(float(set_range_max.get())) * 1000]

    ax.clear()
    ax.set_title("Normal Force")
    ax.set_xlabel("Time(mSec)")
    ax.set_ylabel("Force (nN)")

    ax.grid(b=True, which='major', color='#666666', linestyle='-')
    ax.minorticks_on()
    ax.grid(b=True, which='minor', color='#666666', linestyle='-', alpha=0.2)

    ax2.clear()
    ax2.set_title("Friction Force")
    ax2.set_xlabel("Time(mSec)")
```

```
    ax2.set_ylabel("Force (mV)")

    ax.plot(adhesion_force, color="blue")
    ax2.plot(friction_force, color="red")

    ax2.grid(b=True, which='major', color='#666666', linestyle='-')
    ax2.minorticks_on()
    ax2.grid(b=True, which='minor', color='#666666', linestyle='-', alpha=0.2)
    canvas.draw()

def find_peaks_data():
    global DATA
    filename = input_entry.get()
    alldata = afmreader(filename, verbose=False).data
    # Reading data
    adhesion_data = alldata['Spec']['Pause Forward']['Deflection']
    friction_data = alldata['Spec']['Pause Forward']['Friction force']
    adhesion_force = adhesion_data[0][int(float(set_range_min.get())) *
                                  1000: int(float(set_range_max.get())) * 1000] * 1e9
    friction_force = friction_data[0][int(float(set_range_min.get())) *
                                  1000: int(float(set_range_max.get())) * 1000]

    adhesion_force = adhesion_force - mean(adhesion_force)
    friction_force = friction_force - mean(friction_force)
    if abs(mean(adhesion_force) - max(adhesion_force)) > abs(mean(adhesion_force) -
min(adhesion_force)):
        peak_height_ad = (max(adhesion_force) - mean(adhesion_force)) / 2
    else:
        adhesion_force = -adhesion_force
        peak_height_ad = (max(adhesion_force) - mean(adhesion_force)) / 2

    if abs(mean(friction_force) - max(friction_force)) > abs(mean(friction_force) - min(friction_force)):
        peak_height_fr = (max(friction_force) - mean(friction_force)) / 2
    else:
        friction_force = -friction_force
        peak_height_fr = (max(friction_force) - mean(friction_force)) / 3

    ax.clear()
    ax2.clear()
    peaks_ad, props_ad = find_peaks(adhesion_force, height=peak_height_ad, distance=300,
prominence=peak_height_ad,
                                  width=20)
    ax.plot(adhesion_force, color="blue")
    ax2.plot(friction_force, color="red")
    ax.plot(peaks_ad, adhesion_force[peaks_ad], "x")
```

```
    ax.grid(b=True, which='major', color='#666666', linestyle='-')
    ax.minorticks_on()
    ax.grid(b=True, which='minor', color='#666666', linestyle='-', alpha=0.2)
    ax.set_title("Normal Force")
    ax.set_xlabel("Time(mSec)")
    ax.set_ylabel("Force (nN)")
    ax.vlines(x=peaks_ad, ymin=adhesion_force[peaks_ad] - props_ad["prominences"],
              ymax=adhesion_force[peaks_ad], color="C1")
    ax.hlines(y=props_ad["width_heights"], xmin=props_ad["left_ips"],
              xmax=props_ad["right_ips"], color="C1")

    peaks_fr, props_f = find_peaks(friction_force, height=peak_height_fr, distance=300,
prominence=peak_height_fr,
                                   width=20)
    ax2.plot(peaks_fr, friction_force[peaks_fr], "x")
    ax2.set_title("Friction Force")
    ax2.set_xlabel("Time(mSec)")
    ax2.set_ylabel("Force (mV)")
    ax2.grid(b=True, which='major', color='#666666', linestyle='-')
    ax2.minorticks_on()
    ax2.grid(b=True, which='minor', color='#666666', linestyle='-', alpha=0.2)

    ax2.vlines(x=peaks_fr, ymin=friction_force[peaks_fr] - props_f["prominences"],
               ymax=friction_force[peaks_fr], color="C1")
    ax2.hlines(y=props_f["width_heights"], xmin=props_f["left_ips"],
               xmax=props_f["right_ips"], color="C1")

    DATA = {"Adhesion": {"peak_heights": -adhesion_force[peaks_ad] + props_ad["prominences"],
                         "peak_widths": -props_ad["left_ips"] + props_ad["right_ips"],
                         "time_bn_peaks": append(peaks_ad[1:], 0) - append(peaks_ad[0:-1], 0),
                         "peaks": peaks_ad, },
            "friction": {"peak_heights": -friction_force[peaks_fr] + props_f["prominences"],
                         "peak_widths": -props_f["left_ips"] + props_f["right_ips"],
                         "time_bn_peaks": append(peaks_fr[1:], 0) - append(peaks_fr[0:-1], 0),
                         "peaks": peaks_fr, }}
    canvas.draw()

def plot():
    global DATA
    filename = input_entry.get()
    alldata = afmreader(filename, verbose=False).data
    # Reading data
    adhesion_data = alldata['Spec']['Pause Forward']['Deflection']
    friction_data = alldata['Spec']['Pause Forward']['Friction force']
    adhesion_force = adhesion_data[0][int(float(set_range_min.get())) *
```

```
                                1000: int(float(set_range_max.get())) * 1000] * 1e9
    friction_force = friction_data[0][int(float(set_range_min.get())) *
                                1000: int(float(set_range_max.get())) * 1000]

    plot(adhesion_force)
    show()

def create_window():
    new_window = tk.Toplevel(main_window)
    return new_window

main_window = tk.Tk()
main_window.configure(background='light blue')
main_window.geometry('1000x900')
pw = tk.PanedWindow(main_window, orient='vertical')
main_window.resizable(False, True)
plotting_frame = tk.LabelFrame(pw, text='Real Time', bg='white', width=1000, height=550, bd=5,
relief=tk.SUNKEN)
controls_frame = tk.LabelFrame(pw, text='Controls', background='light grey', width=600, height=150)
output_frame = tk.LabelFrame(pw, text='Output', background='light grey', width=600, height=150)
input_frame = tk.LabelFrame(pw, text='Input', background='light grey', width=600, height=150)
# credit_frame = tk.LabelFrame(pw, text='Thank you!', background='light grey', width=600,
height=50)

controls_frame.grid(column=0, row=1, padx=20, pady=20)
plotting_frame.grid(column=0, row=2, padx=20, pady=20)
output_frame.grid(column=0, row=3, padx=20, pady=20)
input_frame.grid(column=0, row=0, padx=20, pady=20)
# credit_frame.grid(column=0, row=4, padx=20, pady=20)

input_path = tk.Label(input_frame, text="Input File Path:")
input_path.grid(column=0, row=0, padx=10)
input_entry = tk.Entry(input_frame, width=20)
input_entry.grid(column=1, row=0, padx=10)
browse1 = tk.Button(input_frame, text="Browse", command=input_field)
browse1.grid(column=2, row=0, padx=10)
set_range_min = tk.Entry(input_frame, width=10)
set_range_min.grid(column=4, row=0, padx=10)
set_range_max = tk.Entry(input_frame, width=10)
set_range_max.grid(column=5, row=0, padx=10)
range_path = tk.Label(input_frame, text="Set Range (min): [Min]  [Max]")
range_path.grid(column=3, row=0, padx=10)

start_button = tk.Button(controls_frame, text='Start Monitoring', width=20, height=2, borderwidth=3,
                         command=start_plot)
```

```
start_button.grid(row=0, column=0, padx=60)
analyze_button = tk.Button(controls_frame, text='Find Peaks', width=20, height=2, borderwidth=3,
                           command=find_peaks_data)

analyze_button.grid(row=0, column=1, padx=60)

# plot_graphs = tk.Button(controls_frame, text='Plot Graphs', width=20, height=2, borderwidth=3,
#                         command=plot)
# plot_graphs.grid(row=0, column=2, padx=60)

fig = Figure()
fig.set_tight_layout('tight')
ax = fig.add_subplot(211)
ax.set_title("Normal Force")
ax.set_xlabel("Time(mSec)")
ax.set_ylabel("Force (nN)")
ax.set_xlim(0, 30000)
ax.grid(b=True, which='major', color='#666666', linestyle='-')
ax.minorticks_on()
ax.grid(b=True, which='minor', color='#666666', linestyle='-', alpha=0.2)

ax2 = fig.add_subplot(212)
ax2.set_title("Friction Force")
ax2.set_xlabel("Time(mSec)")
ax2.set_ylabel("Force (mV)")
ax2.set_xlim(0, 30000)
ax2.grid(b=True, which='major', color='#666666', linestyle='-')
ax2.minorticks_on()
ax2.grid(b=True, which='minor', color='#666666', linestyle='-', alpha=0.2)

save_button = tk.Button(output_frame, text='Save', width=10, height=2, borderwidth=3,
command=data_automation)
save_button.pack(side=tk.LEFT, padx=26)

exit_button = tk.Button(output_frame, text='Close', width=10, height=2, borderwidth=3,
command=main_window.destroy)
exit_button.pack(side=tk.RIGHT, padx=26)


credit_path = tk.Label(output_frame, text="Created by: Gaitas group @ Mt Sinai", font=("Ariel", 10))
credit_path.pack(side=tk.BOTTOM)

canvas = FigureCanvasTkAgg(fig, master=plotting_frame)
canvas.get_tk_widget().place(x=10, y=0, width=900, height=500)
canvas.draw()
```

```
pw.add(input_frame)
pw.add(controls_frame)
pw.add(plotting_frame)
pw.add(output_frame)
pw.pack(fill='both', expand=True)

main_window.mainloop()
```

Figure S1. Example of the analysis output.

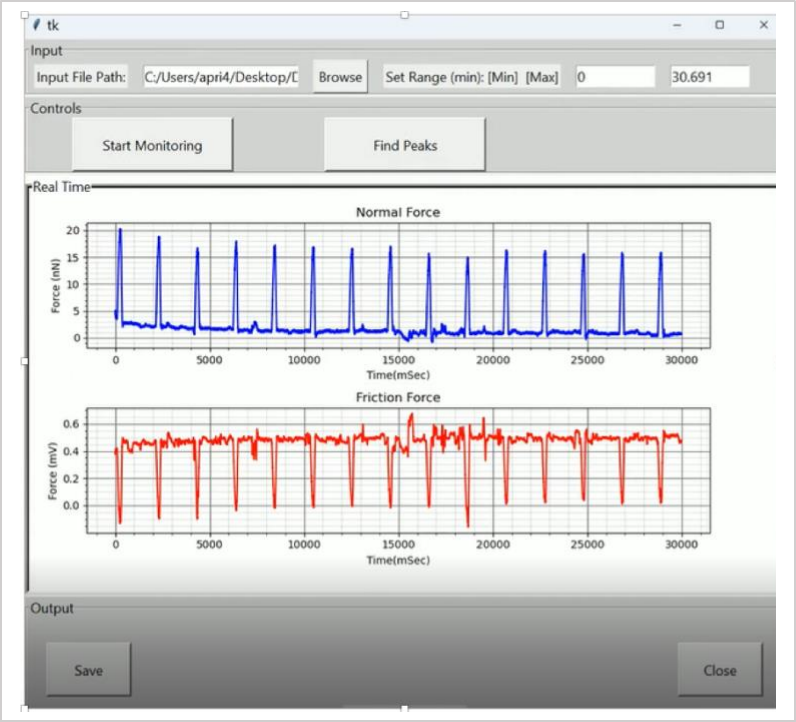

Figure S2. Example of analysis results saved to an Excel file.

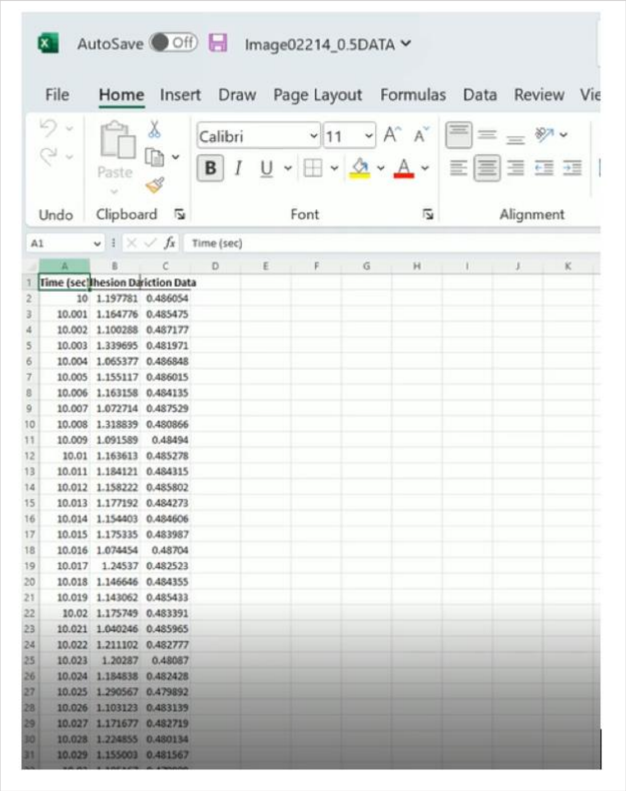

Video S1. AFM cantilever and iPSC-CMs for normal and lateral force measurements. Screen recording of field of view showing AFM cantilever for normal and lateral force measurements and iPSC-CM beating during 0.5 Hz electrical stimulation.
